# Adaptive AOA-Aided TOA Self-Positioning for Mobile Wireless Sensor Networks

**DOI:** 10.3390/s101109742

**Published:** 2010-11-01

**Authors:** Chih-Yu Wen, Fu-Kai Chan

**Affiliations:** Department of Electrical Engineering, Graduate Institute of Communication Engineering, National Chung Hsing University, Taichung 402, Taiwan; E-Mail: 58990006@yahoo.com.tw

**Keywords:** wireless sensor networks, fuzzy control, particle filter, adaptive positioning

## Abstract

Location-awareness is crucial and becoming increasingly important to many applications in wireless sensor networks. This paper presents a network-based positioning system and outlines recent work in which we have developed an efficient principled approach to localize a mobile sensor using time of arrival (TOA) and angle of arrival (AOA) information employing multiple seeds in the line-of-sight scenario. By receiving the periodic broadcasts from the seeds, the mobile target sensors can obtain adequate observations and localize themselves automatically. The proposed positioning scheme performs location estimation in three phases: (I) AOA-aided TOA measurement, (II) Geometrical positioning with particle filter, and (III) Adaptive fuzzy control. Based on the distance measurements and the initial position estimate, adaptive fuzzy control scheme is applied to solve the localization adjustment problem. The simulations show that the proposed approach provides adaptive flexibility and robust improvement in position estimation.

## 1. Introduction

One of the most needed and challenging components in a wireless sensor network is the development of practical localization algorithms for the automatic discovery of sensor position. Robust and distributed internal algorithms of lower cost are required for sensor positioning problems due to the low power of wireless sensor network. Common ranging techniques are receiver signal strength indicator (RSSI), time of arrival (TOA), time difference of arrival (TDOA) and angle of arrival (AOA). For TDOA, centralized sophisticated estimation schemes may lead to computation-intensive problems [1], and in order to suppress the estimation error, a large amount of distance estimates have to be processed for each target node [2,3], which may not be practical in wireless sensor networks. For conventional TOA scheme, at least three TOA measurements must be obtained from three line-of-sight (LOS) seeds (i.e., reference nodes). In order to estimate the position of a moving target sensor in most environments, incorporating angle information may help tackle the localization problem in addition to distance measurements. Thus, an AOA-aided TOA localization scheme may be employed to make the position estimation possible. In general, the localization problem can be solved by the joint AOA/TOA positioning scheme using a single seed [4]. However, in the case of poor observations, more AOA-aided TOA measurements may be applied to complement the measurements of the environment [5].

Due to the propagation environments, some of the propagation paths between the mobile target sensor and the seeds may be non-line-of-sight (NLOS) paths, which have been demonstrated that the NLOS error may degrade the estimation performance and linearly increase the mean location error [6]. Several NLOS mitigation techniques (e.g., the maximum likelihood estimator, least squares techniques) [7–13] have been proposed to solve the location estimation problem in the NLOS scenario such that the NLOS seeds may be first identified and then the target sensor position can be estimated using the LOS seeds.

With the NLOS mitigation techniques described above, here we introduce an AOA/TOA hybrid self-positioning scheme, the *AOA-Aided TOA Positioning Algorithm* (ATPA) and present a network-based positioning system considering the relative movements between the multiple seeds and the mobile sensor. The main assumptions are: (1) The clocks of the seeds and the mobile sensors with unknown positions are synchronized; (2) The target sensor will not dramatically change its moving direction; (3) The seeds broadcast their position information periodically. The goal of the proposed scheme is to estimate the target position coincided with the broadcasting time stamp of the seeds. Accordingly, the ATPA positioning scheme performs location estimation in three phases: (I) AOA-Aided TOA Measurement, (II) Geometrical Positioning with Particle Filtering, and (III) Adaptive Fuzzy Control.

In Phase I, since the movement of a target sensor introduces differences between arrival times of the seeds, the aided AOA information may be used to modify the TOA measurements, which can be applied to compute the location estimates. In Phase II, the Bayesian particle filter [14] is used to estimate the unknown sensor position from state equations. The objective is to find feasible position to make the error of state vector minimum. After obtaining the initial position estimate, the localization adjustment problem can be solved by applying the operations of Phase III (adaptive fuzzy control). Here, we define the procedures of adaptive fuzzy control in three steps: (I) Determining fuzzy controller input variables, (II) Applying the gradient descent learning [15] and constructing adaptive fuzzy rules, and (III) doing defuzzification.

The major contributions and key features of this paper are: (1) The operation of the proposed ATPA can be regarded as the reverse operation of TDOA, which allows all mobile sensors to obtain adequate observations and to perform self-localization by receiving the signals from the seeds without interfering with each other. Therefore, compared with conventional TDOA approaches, the purpose of energy conservation can be achieved since the proposed method involves effective communication between the seeds and the target sensors with less communication overhead. Moreover, a modification scheme of distance measurement is proposed to coordinate the signals and information in a scenario with multiple seeds; (2) One of the main advantages of particle filtering method is that the mobile sensor carries along a complete distribution of estimates of its position. Thus, the distribution is inherently a measure of the accuracy of the positioning system; (3) Due to the characteristics of the learning process for tuning fuzzy rules, the proposed ATPA approach owns adaptive flexibility when dealing with uncertainty in position estimation.

This paper is organized as follows: Section 2 reviews the literatures on hybrid TOA/AOA positioning schemes and position refinement techniques. Section 3 formulates the position estimation problem and derives an adaptive self-localization solution that relies on a distributed positioning protocol [16]. Section 4 presents an estimation-theoretic analysis of the proposed measurement mechanisms to assess the achievable estimation accuracy. Two main positioning errors are considered: (1) the distance-dependent positioning error and (2) the angle-dependent positioning error. These two positioning errors are examined carefully to assess their impacts on the positioning accuracy. In Section 5, with a number of sensible settings, the feasibility of the proposed schemes is examined via simulation and numerical results. The final section makes a conclusion and shows future research directions.

## 2. Literature Review

Mobile location with TOA/AOA information at a single base station is first proposed in [4]. The authors in [17] analyze the location accuracy of an TOA/AOA hybrid algorithm with a single base station in the LOS scenario. Deng and Fan [5] introduce an TOA/AOA location algorithm with multiple base stations. However, the speed of the mobile station is assumed to be very low and the relative movement between the base station and the mobile station is not considered. [18] utilizes a constrained nonlinear optimization approach, when range measurements are available from three base stations only. Bounds on the non-line-of-sight (NLOS) error and the relationship between the true ranges are extracted from the geometry of the cell layout and the measured range circles to serve as constraints. [19] introduces two hybrid TOA/AOA techniques, Enhanced Time of Arrivals (E-TOA) and Enhanced Angle of Arrival (E-AOA), in order to optimize the location positioning estimations. [20] proposes a residual test (RT) that can simultaneously determine the number of LOS base stations and identify them such that localization can proceed with only those LOS base stations. Hybrid location methods by combining time and angle measurements can reduce the number of receiving base stations and improve the coverage of location-based service simultaneously. Comprehensive surveys of design challenges and recently proposed hybrid positioning algorithms for wireless networks can be found in [7,21–24].

Due to the error caused by the location estimation algorithm (the estimation error) and the error intrinsic to the problem (noisy distance measurements), location adjustment algorithms are needed in order to improve the estimation accuracy. There are several refinement schemes that have been proposed in the literature. Since the particle filter looses diversity in the samples, a sequential Monte Carlo framework [25] can be used to generate new samples and provide improved estimation accuracy (e.g., random walk Monte Carlo methods, Metropolis-Hastings (M-H) algorithm [26]). The basic idea is to simulate an ergodic Markov chain such that the new samples are asymptotically distributed based on the target probability distribution. Thus, applying the Markov chain Monte Carlo (MCMC) method on each estimated sensor right after the location estimation allows estimation error to be reduced in a distributed way. Based on the sequential Monte Carlo framework, the MCMC method can be applied directly to evaluate the most expectation of target position. In Section 5, the performance of the MCMC method is explored to provide comprehensive performance assessment of the proposed adaptive fuzzy control approach in Phase III.

[27] attempts to find locations for the sensors which best fit the set of all range measurements made in the network in a least-mean-squares sense. [28] demonstrates the utility of nonparametric belief propagation (NBP) for self-localization in sensor networks. However, the computational complexity and communication costs inherent in a distributed implementation of NBP are high. [29] presents a localization algorithm based on a spring model (LASM) method to simulates the dynamics of physical spring system and to estimate the positions of nodes. Simulation results show that the LASM method reduces the computational complexity, while maintaining the localization accuracy. [30] presents the collaborative multilateration to enable ad-hoc deployed sensor nodes to accurately estimate their locations by using known beacon locations that are several hops away and distance measurements to neighboring nodes. To prevent error accumulation in the network, node locations are computed by setting up and solving a global non-linear optimization problem. [31] presents an approach called AHLoS (Ad-Hoc Localization System) that enables sensor nodes to discover their locations using a set distributed iterative algorithms. [32] proposes a heuristic refinement approach to improve position estimates. [33] proposes an iterative quality-based localization (IQL) algorithm for location discovery. The IQL algorithm first determines an initial position estimate, after which the Weighted Least-Squares (WLS) algorithm is used iteratively to refine the position. In the WLS algorithm the Gaussian distribution is used to determine the reliability of measurements.

Instead of applying the above refinement approaches, the position estimation problem can be solved with fuzzy logic algorithms as well. [34] presents a swarm-based fuzzy logic control (FLC) mobile sensor network approach for collaboratively locating the hazardous contaminants in an unknown large-scale area, which maintains a stable communication network for collaborative exploration and information fusion. [35] proposes a fixed fuzzy control scheme (FLAME algorithm) for minimizing the localization error. FLAME algorithm works very well in estimating error-free locations. However, given a poor initial estimate, the fuzzy logic controller has limited capability to improve the accuracy. Moreover, a equalizer is needed to make further corrections to fuzzy estimate, which may lead to extra computational cost. Since only using fixed fuzzy decision rules [35] may not be able to provide accurate estimates, in this paper, we refer to the concept described in [15] and develop an adaptive fuzzy control system, which tunes the fuzzy rules without changing the form of the fuzzy rule table used in fuzzy controls and trains system parameters based on the gradient descent method to minimize the position error under the circumstance of measurement uncertainties. The performance comparison of the FLAME algorithm and the proposed ATPA approach are presented in Section 5.

## 3. Principles of Adaptive AOA-Aided TOA Positioning

**Figure 1.**
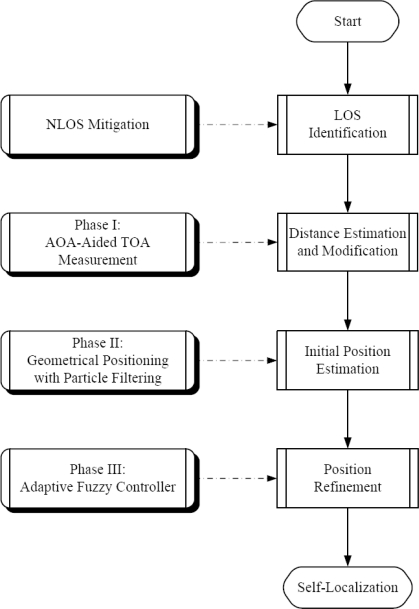
Illustration of block diagram for the ATPA method.

This section describes an adaptive distributed algorithm for positioning the mobile sensors. Figure 1 shows the block diagrams for the proposed localization system. The main steps for distributed self-positioning are LOS identification, distance measurement and modification, initial position estimation, and estimation refinement, which are achieved by applying NLOS mitigation techniques [7–13], AOA-aided TOA information, geometric localization using particle filter, and adaptive fuzzy control scheme, respectively. Assuming that the LOS seeds are identified, the following subsections detail the operations and design principles of the proposed ATPA approach.

### 3.1. AOA-Aided TOA Measurement

**Figure 2.**
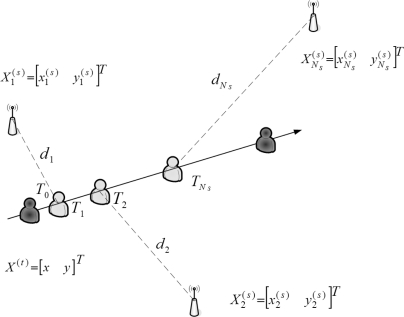
Differences between signal arrival time stamps.

**Figure 3.**
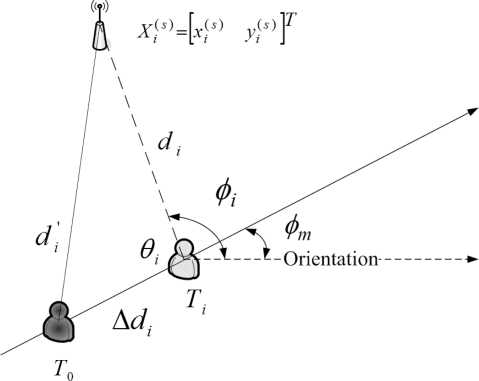
The concept of measurement modification.

As shown in Figure 2, due to the locations of the seeds, the time stamps of the received signals from the seeds are different. Thus, a measurement modification may be applied to coordinate the signals and information in a scenario with multiple seeds. Assume that the unknown sensors are aware of their orientations before the estimation. Figure 3 depicts the concept of measurement modification. Let *T*_0_ be the broadcasting time of the seed. Let *T_i_* be the time stamp of the received signal from Seed *i*. Denote *d_i_* as the distance between Seed *i* and the target sensor. Denote Δ*d_i_* as the movement of the target sensor from time stamp *T*_0_ to time stamp *T_i_*. Denote *φ_i_* as the direction of the received signal from Seed *i* with respect to the orientation. Denote *φ_m_* as the moving direction of the target sensor with respect to the orientation.

Hence, based on *d_i_*, Δ*d_i_*, the AOA information, and the information of mobility model (e.g., the moving direction), the distance measurement may be modified by

(1) $$d_{i}^{\prime 2}=d_{i}^{2}+\Delta d_{i}^{2}-2d_{i}\Delta d_{i}\text{ cos}(\theta_{i})$$

Note that because of the locations of the seeds and the target sensor, the decision criterion of the angle *θ_i_* yields

(2) $$\theta_{i}=\{\begin{array}{ll} \pi -||\phi_{m}\left|-\right|\phi_{i}||, & \text{for }\;(\phi_{m}\cdot \phi_{i})\geq 0\\ \pi -||\phi_{m}\left|+\right|\phi_{i}||, & \text{otherwise} \end{array}$$

**Figure 4.**
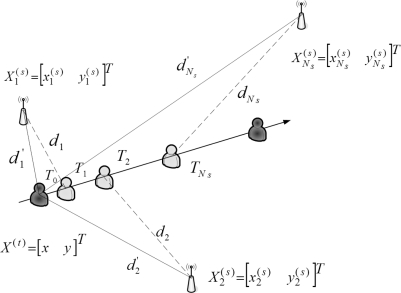
AOA-Aided TOA measurements with multiple seeds.

Accordingly, Figure 4 shows that given *N_s_* seeds, the *N_s_* modified distance measurements may be applied to estimate the location of the target sensor at time *T*_0_.

### 3.2. Geometrical Positioning with Particle Filtering

Suppose that a mobile sensor does not know its position but is able to receive information from neighboring seeds which are assumed to have accurate position information. There are many ways to ‘solve’ this location problem. This section details the Bayesian particle filter method which may be preferred because it is robust to noisy measurements, it allows for flexible information transmission, and it can be robust to lost or lossy data.

#### 3.2.1. The Measurement Term

Assume the target sensor *m* obtains the modified distance measurements (*i.e.*, the AOA-aided TOA measurements) from neighboring seeds and estimates its own position using the particle filter. The position of the target sensor is given by the discrete-time state equation

(3) $$x_{k}=\Phi x_{k-1}+\Gamma \lambda_{k}$$

where *x_k_* is the position of the mobile sensor and λ*_k_* is an uncorrelated Gaussian diffusion term describing the uncertainty. Note that this system equation is suitable for many different systems and the only changes will be the matrixes Φ and Γ, which depend on the system model.

The measurement term for the target sensor *m* is

(4) $$z_{k}=\sum_{\ell \in H_{m}}\left|\left|x_{k}^{m}-x_{k}^{\ell }\right|-d_{m\ell }\right|+v_{k}$$

where the sum is over the nearby seeds with location $x_{k}^{\ell }$, *d_mℓ_* represents the measured distance between the estimated target sensor *m* and Seed *ℓ* and may be approximated in application by the inverse of the signal strength or by calculated from the time delay between transmission and reception [36]; the measurement noise is another uncorrelated zero mean Gaussian white noise process; the set *H_m_* is the chosen seeds for the measurement.

#### 3.2.2. Geometrical Positioning

Particle filter is an algorithm of estimation used to estimate the unknown sensor position from state equations. The objective is to find feasible position to make the error of state vector *x* minimum. The state vector is represented as a set of random samples updated and propagated with the algorithm. One of the main advantages of this approach is that the mobile sensor carries along a complete distribution of estimates of its position. Therefore, the distribution is inherently a measure of the accuracy of the positioning system—hence, if a given task requires a certain accuracy, it is possible to determine if that level of accuracy is currently available. Moreover, [37] presents a case study of applying particle filters to location estimation for ubiquitous computing. Therefore, our approach may be computationally affordable by sensor nodes. The particle filter method is shown in Table 1.

### 3.3. Adaptive Fuzzy Control

Due to the effectiveness of the fuzzy logic controller in minimizing the estimation error [15,35], an adaptive fuzzy control system is developed to approach the true target coordinate.

#### 3.3.1. Inputs of the Fuzzy Control

**Figure 5.**
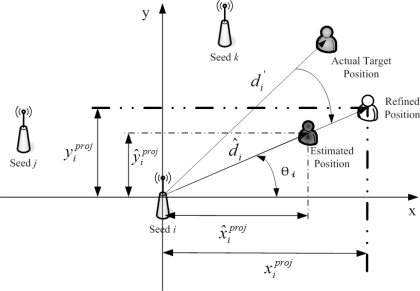
Estimation adjustment without AOA information.

In the proposed fuzzy control system, two scenarios are considered. For Scenario 1, as depicted in Figure 5, the target sensor has no AOA information. Given the initial estimate of target position in Phase II and the reference position of Seed *i*, the vector ${\vec{\hat{d}}}_{i}$ and angle *θ_i_* (in radians) are obtained. Hence, the projections of ${\vec{\hat{d}}}_{i}$ onto x-axis and y-axis are

(5) $${\hat{x}}_{i}^{\mathrm{proj}}={\hat{d}}_{i}\cdot \text{ cos}(\theta_{i}),\;{\hat{y}}_{i}^{\mathrm{proj}}={\hat{d}}_{i}\cdot \text{ sin}(\theta_{i})$$

Instead of using the estimated target position, the modified distance measurement (*i.e.*, ${\vec{d}}_{i}^{\prime }$) and angle *θ_i_* are applied to find the projections, which are

(6) $$x_{i}^{\mathrm{proj}}=d_{i}^{\prime }\cdot \text{ cos}(\theta_{i}),\;y_{i}^{\mathrm{proj}}=d_{i}^{\prime }\cdot \text{ sin}(\theta_{i})$$

**Figure 6.**
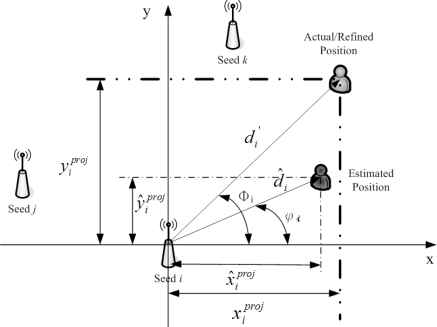
Estimation adjustment with AOA information.

For Scenario 2, as shown in Figure 6, the target sensor has received AOA information. Following the same operations above, we have

(7) $${\hat{x}}_{i}^{\mathrm{proj}}={\hat{d}}_{i}\cdot \text{ cos}(\varphi_{i}),\;{\hat{y}}_{i}^{\mathrm{proj}}={\hat{d}}_{i}\cdot \text{ sin}(\varphi_{i})$$

(8) $$x_{i}^{\mathrm{proj}}=d_{i}^{\prime }\cdot \text{ cos}\left(\Phi_{i}\right),\;y_{i}^{\mathrm{proj}}=d_{i}^{\prime }\cdot \text{ sin}\left(\Phi_{i}\right)$$

Note that angle Φ*_i_* is captured from the AOA information and the information of mobility model, and angle φ*_i_* is derived from the initial target position estimate and the reference position of Seed *i*. Accordingly, $x_{i}^{\mathrm{diff}}={\hat{x}}_{i}^{\mathrm{proj}}-x_{i}^{\mathrm{proj}}$ and $y_{i}^{\mathrm{diff}}={\hat{y}}_{i}^{\mathrm{proj}}-y_{i}^{\mathrm{proj}}$ are fed as inputs to the fuzzy control system for the two scenarios described above.

#### 3.3.2. Gradient Descent Learning

The proposed fuzzy control system uses fuzzy logic and gradient descent method to adjust a suitable answer for the target sensor position. The objective function to be minimized is defined by

(9) $$E=\frac{1}{2}{\left(u-u^{d}\right)}^{2}$$

where *u^d^* is a desired output value for an input vector, and *u* is a fuzzy inference value. Here, we use the projections onto the x-axis of ${\vec{\hat{d}}}_{i}$ and ${\vec{d}}_{i}^{\prime }$ to explain the operation of the fuzzy system and minimize the objective function *E*. In this case, the desired output value for input vector x = [*x*_1_, *x*_2_, . . ., *x_L_*]*^T^* is $u_{i}^{d}=x_{i}^{\mathrm{proj}}$ and the fuzzy inference value is $u_{i}={\hat{x}}_{i}^{\mathrm{proj}}+\delta$. Therefore, *E* can be rewritten by

(10) $$E=\sum_{i=1}^{L}\frac{1}{2}{\left(u_{i}-u_{i}^{d}\right)}^{2}$$

(11) $$=\sum_{i=1}^{L}\frac{1}{2}{\left[({\hat{x}}_{i}^{\mathrm{proj}}+\delta )-x_{i}^{\mathrm{proj}}\right]}^{2}$$

(12) $$=\sum_{i=1}^{L}\frac{1}{2}{\left[\left(x_{i}^{\mathrm{diff}}+\delta \right)\right]}^{2}$$

where *δ* is the output of fuzzy membership

(13) $$\delta =\frac{\sum_{j=1}^{r}q_{j}(\text{x})w_{j}}{\sum_{j=1}^{r}q_{j}(\text{x})}$$

which is calculated by center of area (COA) method [38]. Note that

(14) $$q_{j}(\text{x})=\prod_{i=1}^{L}\mu_{A_{i}^{j}}(x_{i}),\;j=1,2,\ldots ,r$$

is the defined firing strength of rule *j, r* is the number of fuzzy rules, $\mu_{A_{i}^{j}}$ is the membership function of the precondition part, *w_j_* is the training parameter, and the *x*_1_, *x*_2_, . . ., *x_L_* are input variables.

Suppose $\mu_{A_{i}^{j}}$ is a Gaussian membership function for input variable *x_i_* of rule *j*. Thus, *E* is further given by

(15) $$E=\sum_{i=1}^{L}\frac{1}{2}{\left[\left(x_{i}^{\mathrm{diff}}+\frac{\sum_{j=1}^{r}q_{j}(\text{x})w_{j}}{\sum_{j=1}^{r}q_{j}(\text{x})}\right)\right]}^{2}$$

(16) $$=\sum_{i=1}^{L}\frac{1}{2}{\left[(x_{i}^{\mathrm{diff}}+\frac{\sum_{j=1}^{r}\prod_{i=1}^{L}\mu_{A_{i}^{j}}\left(x_{i}\right)w_{j}}{\sum_{j=1}^{r}\prod_{i=1}^{L}\mu_{A_{i}^{j}}}\right]}^{2}$$

with

(17) $$\mu_{A_{i}^{j}}=\text{ exp }\left[-\frac{{\left(x_{i}-m_{i}^{j}\right)}^{2}}{{\left(\sigma_{i}^{j}\right)}^{2}}\right]$$

where *i* = 1, 2, . . ., *L*, *j* = 1, 2, . . ., *r*, and $m_{i}^{j}$ and $\sigma_{i}^{j}$ are the mean and the standard deviation of $\mu_{A_{i}^{j}}$, respectively.

In order to minimize the function *E* and find a better feasible estimate, the training parameters are derived based on gradient descent method [15]. For $m_{i}^{j}$, we have

(18) $$m_{i}^{j}(t+1)=m_{i}^{j}(t)-\eta \frac{\partial E}{\partial m_{i}^{j}}$$

(19) $$=m_{i}^{j}(t)-\eta \sum_{i=1}^{L}\left[(x_{i}^{\mathrm{diff}}+\delta )\right]\cdot \frac{w_{j}-\delta }{\sum_{K=1}^{r}q_{K}}\cdot q_{j}\cdot \frac{2(x_{i}-m_{i}^{j})}{{\left(\sigma_{i}^{j}\right)}^{2}}$$

where *i* = 1, 2, . . ., *L*, *j* = 1, 2, . . ., *r*, and *η* is the constant step size (0 < *η* < 1). Similarly, the training process of $\sigma_{i}^{j}$ is given by

(20) $$\sigma_{i}^{j}(t+1)=\sigma_{i}^{j}(t)-\eta \frac{\partial E}{\partial \sigma_{i}^{j}}$$

(21) $$=\sigma_{i}^{j}(t)-\eta \sum_{i=1}^{L}\left[(x_{i}^{\mathrm{diff}}+\delta )\right]\cdot \frac{w_{j}-\delta }{\sum_{K=1}^{r}q_{K}}\cdot q_{j}\cdot \frac{2{\left(x_{i}-m_{i}^{j}\right)}^{2}}{{\left(\sigma_{i}^{j}\right)}^{3}}$$

and the training parameter *w_j_* yields

(22) $$w_{j}(t+1)=w_{j}(t)-\eta \frac{\partial E}{\partial w_{j}}$$

(23) $$=w_{j}(t)-\eta \sum_{i=1}^{L}\left[(x_{i}^{\mathrm{diff}}+\delta )\right]\cdot \frac{q_{j}}{\sum_{K=1}^{r}q_{K}}.$$

#### 3.3.3. Constructing Adaptive Fuzzy Rules

**Figure 7.**
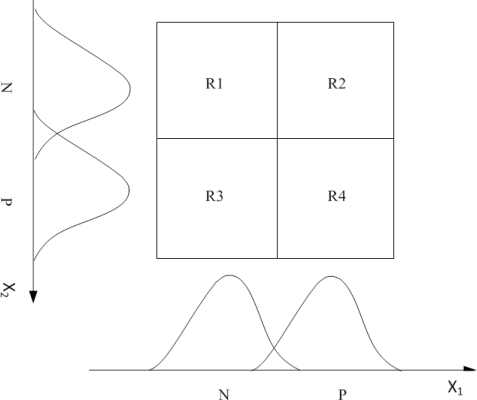
Gaussian membership functions for the linguistic states.

Based on the inputs as detailed in Section 3.3.1, the linguistic variables used for the input of the fuzzy logic controller system are N (negative) and P (positive). Gradient descent method is used to decide linguistic variables of N and P, which represents a measure of the difference between ${\hat{x}}_{i}^{\mathrm{proj}}\left({\hat{y}}_{i}^{\mathrm{proj}}\right)$ and $x_{i}^{\mathrm{proj}}\left(y_{i}^{\mathrm{proj}}\right)$. In Figure 7, Gaussian membership functions are developed for the linguistic states. In this work, four fuzzy rules are developed and the training parameters ( $m_{j}^{i}$, $\sigma_{j}^{i}$, *w_j_*) are calculated by the above gradient descent method. Table 2 expresses the fuzzy logic in terms of fuzzy IF-THEN rules, which implements mapping of input functions into output functions.

#### 3.3.4. Defuzzification Method

There are many methods available for doing defuzzification (e.g., Center of Area, Mean Maximum). Here, we use the Center of Area (COA) method to determine the defuzzification value for the x-coordinate. Note that the above operations are for the refinement of the x-coordinate on x-axis. Similar procedures can be performed for the y-coordinate on the y-axis.

## 4. Analysis of Positioning Accuracy

Referring to [39], evaluating the computation process and the significance of approximate accuracy is an important step in deriving either exact or approximate solutions for the localization problem. This section presents an estimation-theoretic analysis of the proposed measurement mechanisms to assess the achievable estimation accuracy.

### 4.1. CRLB of TDOA

The location geometry is shown in Figure 2, where *N_s_* seeds at locations ${\text{x}}_{i}^{\left(s\right)}={\left(x_{i}^{\left(s\right)},y_{i}^{\left(s\right)}\right)}^{T}$ are use to locate a target at position **x**^(*t*)^ = (*x, y*)*^T^* through TDOA measurements. Let *d_i_* be the true distance between the target and Seed *i*,

(24) $$d_{i}=\sqrt{{\left(x-x_{i}^{\left(s\right)}\right)}^{2}+{\left(y-y_{i}^{\left(s\right)}\right)}^{2}}$$

(25) $$u_{i}=d_{i+1}-d_{1}$$

where *i* = 1, 2, . . ., *N_s_* − 1. Assume that

(26) $$r[i]=d_{i}+n_{i}$$

where *n_i_* is the TOA noise that is assumed to be zero-mean Gaussian with variance σ^2^. Thus, the distribution of *r*[*i*] is

(27) $$f(r[i];x,y)=\frac{1}{{\left(2\pi \sigma^{2}\right)}^{N_{s}/2}}\text{exp}(-\frac{\sum_{i=1}^{N_{s}}{\left(r[i]-u_{i}\right)}^{2}}{2\sigma^{2}})$$

where *i* = 1, 2, . . ., *N_s_* and −∞ ≤ *r*[*i*] = ∞.

The CRLB is the lowest possible variance that an unbiased linear estimator can achieve. It is given by the inverse of the Fisher information matrix *I*(x^(*t*)^) defined as

(28) $$I({\text{x}}^{\left(t\right)})=\left[\begin{array}{ll} I_{1,1} & I_{1,2}\\ I_{2,1} & I_{2,2} \end{array}\right]$$

where

(29) $$I_{1,1}=\frac{\partial D^{T}}{\partial x}\Sigma^{-1}\frac{\partial D}{\partial x}$$

(30) $$I_{1,2}=\frac{\partial D^{T}}{\partial x}\Sigma^{-1}\frac{\partial D}{\partial y}$$

(31) $$I_{2,1}=\frac{\partial D^{T}}{\partial y}\Sigma^{-1}\frac{\partial D}{\partial x}$$

(32) $$I_{2,2}=\frac{\partial D^{T}}{\partial y}\Sigma^{-1}\frac{\partial D}{\partial y}$$

with

$$\begin{array}{lll} \frac{\partial D}{\partial x} & = & {\left[\frac{\partial u_{1}(x,y)}{\partial x}\frac{\partial u_{2}(x,y)}{\partial x}\cdots \frac{\partial u_{N_{s}-1}(x,y)}{\partial x}\right]}^{T}\\ & = & {\left[\frac{x-x_{2}^{\left(s\right)}}{d_{2}}-\frac{x-x_{1}^{\left(s\right)}}{d_{1}}\cdots \frac{x-x_{N_{s}}^{\left(s\right)}}{d_{N_{s}}}-\frac{x-x_{1}^{\left(s\right)}}{d_{1}}\right]}^{T} \end{array}$$

$$\begin{array}{lll} \frac{\partial D}{\partial y} & = & {\left[\frac{\partial u_{1}(x,y)}{\partial y}\frac{\partial u_{2}(x,y)}{\partial y}\cdots \frac{\partial u_{N_{s}-1}(x,y)}{\partial y}\right]}^{T}\\ & = & {\left[\frac{y-y_{2}^{\left(s\right)}}{d_{2}}-\frac{y-y_{1}^{\left(s\right)}}{d_{1}}\cdots \frac{y-y_{N_{s}}^{\left(s\right)}}{d_{N_{s}}}-\frac{y-y_{1}^{\left(s\right)}}{d_{1}}\right]}^{T} \end{array}$$

(33) $$\Sigma^{-1}=\frac{1}{\sigma^{2}}\left[\begin{array}{lll} 1 & \cdots & 0\\ \vdots & \ddots & \vdots \\ 0 & \cdots & 1 \end{array}\right].$$

Therefore, the Cramer-Rao bound can then be written as

(34) $$\mathrm{Var}({\text{x}}^{\left(t\right)})\geq I^{-1}({\text{x}}^{\left(t\right)})=\left[\begin{array}{ll} I_{1,1}^{\prime } & I_{1,2}^{\prime }\\ I_{2,1}^{\prime } & I_{2,2}^{\prime } \end{array}\right]$$

Then

(35) $$\mathrm{Var}(\tilde{x})\geq I_{1,1}^{\prime },\;\mathrm{Var}(\tilde{y})\geq I_{2,2}^{\prime }$$

where *I*′_1,1_ is the CRLB of *x* and *I*′_2,2_ is the CRLB of *y*, and the trace of CRLB is the minimum possible target location MSE that any linear unbiased estimator can achieve.

### 4.2. CRLB of TOA

Given the condition probability density function from (27), explicit expressions for the elements of the Fisher information matrix (FIM) can be derived, which yields [20]

(36) $$I({\text{x}}^{\left(t\right)})=\frac{1}{\sigma^{2}}\left[\begin{array}{cc} \sum_{i=1}^{N_{s}}\frac{{\left(x-x_{i}\right)}^{2}}{d_{i}^{2}} & \sum_{i=1}^{N_{s}}\frac{\left(x-x_{i})(y-y_{i}\right)}{d_{i}^{2}}\\ \sum_{i=1}^{N_{s}}\frac{\left(x-x_{i})(y-y_{i}\right)}{d_{i}^{2}} & \sum_{i=1}^{N_{s}}\frac{{\left(y-y_{i}\right)}^{2}}{d_{i}^{2}} \end{array}\right]$$

and the Cramer-Rao lower bound can then be written as

(37) $$\mathrm{Var}({\text{x}}^{\left(t\right)})\geq I^{-1}({\text{x}}^{\left(t\right)})=\left[\begin{array}{cc} I_{1,1}^{\prime } & I_{1,2}^{\prime }\\ I_{2,1}^{\prime } & I_{2,2}^{\prime } \end{array}\right]$$

Note that *I*′_1,1_ and *I*′_2,2_ are CRLBs of *x̂* and *ŷ*, which are diagonal elements of the inverse of the FIM matrix.

### 4.3. CRLB of Joint TOA/AOA

The measurements at the targeted sensor can be modeled as

(38) $$\hat{\tau }=\tau +\delta_{\tau }$$

(39) $$\hat{\phi }=\phi +\delta_{\phi }$$

where *τ* is the true propagation time and *φ* is the true angle information. Note that *δ_τ_* and *δ_φ_* are uncorrelated Gaussian noises with the distributions $\delta_{\tau }∼𝒩(0,\sigma_{\tau }^{2})$ and $\delta_{\phi }∼𝒩(0,\sigma_{\phi }^{2})$. Assuming that the direct path exists between the seed and the target sensor, the estimated position is given by

(40) $$\hat{x}=x_{s}+\upsilon \hat{\tau }\text{ cos}(\hat{\phi })=x_{s}+\hat{r}\text{ cos}(\hat{\phi })$$

(41) $$\hat{y}=y_{s}+\upsilon \hat{\tau }\text{ sin}(\hat{\phi })=y_{s}+\hat{r}\text{ sin}(\hat{\phi })$$

where *r̂* is the distance measurement (*i.e.*, *r̂* = *υτ̂= r* + *υδ_τ_*), (*x_s_, y_s_*) is the true position of the seed and *v* is the speed of signal. Assuming *δ_τ_* and *δ_φ_* are sufficiently small, the variance of the position estimation *p̂* is approximated by

(42) $$\sigma_{p}^{2}\approx \upsilon^{2}\sigma_{\tau }^{2}+d^{2}\sigma_{\phi }^{2}=\sigma_{r}^{2}+d^{2}\sigma_{\phi }^{2}$$

Given the above assumptions [17], the CRLBs with single seed and multiple seeds are derived as follows, respectively.

#### 4.3.1. Single Seed

The probability density function of g = [*r̂*, *φ̂*] is

(43) $$\begin{array}{c} f(\text{g};x,y)=\frac{1}{\sqrt{2\pi \sigma_{r}^{2}}}\cdot \text{ exp }\left[-\frac{1}{2\sigma_{r}^{2}}{\left(\hat{r}-d\right)}^{2}\right]\\ \cdot \frac{1}{\sqrt{2\pi \sigma_{\phi }^{2}}}\cdot \text{ exp }\left[-\frac{1}{2\sigma_{\phi }^{2}}{\left(\hat{\phi }-\text{ arctan }\left(\frac{y-y_{s}}{x-x_{s}}\right)\right)}^{2}\right] \end{array}$$

Thus, the Fisher information matrix yields

(44) $$I({\text{x}}^{\left(t\right)})=\left[\begin{array}{cc} \frac{{\text{cos}}^{2}(\phi )}{\sigma_{r}^{2}}+\frac{{\text{sin}}^{2}(\phi )}{d^{2}\sigma_{\phi }^{2}} & \frac{\text{sin}(2\phi )}{2}\left[\frac{1}{\sigma_{r}^{2}}-\frac{1}{d^{2}\sigma_{\phi }^{2}}\right]\\ \frac{\text{sin}(2\phi )}{2}\left[\frac{1}{\sigma_{r}^{2}}-\frac{1}{d^{2}\sigma_{\phi }^{2}}\right] & \frac{{\text{sin}}^{2}(\phi )}{\sigma_{r}^{2}}+\frac{{\text{cos}}^{2}(\phi )}{d^{2}\sigma_{\phi }^{2}} \end{array}\right]$$

and the Cramer-Rao bound can then be written as

(45) $$\mathrm{Var}({\text{x}}^{\left(t\right)})\geq I^{-1}({\text{x}}^{\left(t\right)})=\left[\begin{array}{ll} I_{1,1}^{\prime } & I_{1,2}^{\prime }\\ I_{2,1}^{\prime } & I_{2,2}^{\prime } \end{array}\right]$$

Thus,

(46) $$\mathrm{Var}(\tilde{x})\geq I_{1,1}^{\prime },\;\mathrm{Var}(\tilde{y})\geq I_{2,2}^{\prime }$$

#### 4.3.2. Multiple Seeds

Referring to the concept of measurement modification as shown in Figure 3 and normal approximation [40], the modified distance estimate $\hat{d_{i}^{\prime }}$ in (1) may be approximated by

(47) $$\hat{d_{i}^{\prime }}∼{𝒩}_{\int }(\mu_{d_{i}^{\prime }},\sigma_{d_{i}^{\prime }}^{2})$$

with

(48) $$\mu_{d_{i}^{\prime }}=d_{i}^{\prime }=\sqrt{d_{i}^{2}+\Delta d_{i}^{2}-2d_{i}\Delta d_{i}\text{ cos}(\theta_{i})}$$

(49) $$\sigma_{d_{i}^{\prime }}^{2}\approx (1+4{\text{ cos}}^{2}(\theta_{i})\Delta d_{i}^{2})\sigma_{p_{i}}^{2}+(1+4{\text{ cos}}^{2}(\theta_{i})d_{i}^{2})\sigma_{\Delta p_{i}}^{2}+4{\text{ sin}}^{2}(\theta_{i})d_{i}^{2}\Delta d_{i}^{2}\sigma_{\theta_{i}}^{2}$$

where

(50) $$\sigma_{p_{i}}^{2}\approx \sigma_{d_{i}}^{2}+d_{i}^{2}\sigma_{\phi_{i}}^{2}$$

(51) $$\sigma_{\Delta p_{i}}^{2}\approx \sigma_{\Delta d_{i}}^{2}+\Delta d_{i}^{2}\sigma_{\phi_{m}}^{2}$$

(52) $$\sigma_{\theta_{i}}^{2}\approx \sigma_{\phi_{i}}^{2}+\sigma_{\phi_{m}}^{2}$$

Note that *d_i_*, Δ*d_i_, θ_i_, φ_i_,* and *φ_m_* are the estimated parameters with respect to Seed *i* assumed to be Gaussian distributions centered at their true values. Therefore, the distribution of *r*[*i*] is

(53) $$f(r[i];x,y)=\frac{1}{{\left(2\pi \sigma_{i}^{2}\right)}^{N_{s}/2}}\text{ exp}(-\frac{\sum_{i=1}^{N_{s}}{\left(r[i]-d_{i}^{\prime }\right)}^{2}}{2\sigma_{i}^{2}})$$

where *i* = 1, 2, . . ., *N_s_* and −∞ ≤ *r*[*i*] ≤ ∞. Similarly, following the definitions (28) ∼ (32) in Section 4.1 with

$$\begin{array}{lll} \frac{\partial D}{\partial x} & = & {\left[\frac{\partial d_{1}^{\prime }(x,y)}{\partial x}\frac{\partial d_{2}^{\prime }(x,y)}{\partial x}\cdots \frac{\partial d_{N_{s}}^{\prime }(x,y)}{\partial x}\right]}^{T}\\ & = & {\left[\frac{x-x_{1}^{\left(s\right)}}{d_{1}^{\prime }}\frac{x-x_{2}^{\left(s\right)}}{d_{2}^{\prime }}\cdots \frac{x-x_{N_{s}}^{\left(s\right)}}{d_{N_{s}}^{\prime }}\right]}^{T} \end{array}$$

$$\begin{array}{lll} \frac{\partial D}{\partial y} & = & {\left[\frac{\partial d_{1}^{\prime }(x,y)}{\partial y}\frac{\partial d_{2}^{\prime }(x,y)}{\partial y}\cdots \frac{\partial d_{N_{s}}^{\prime }(x,y)}{\partial y}\right]}^{T}\\ & = & {\left[\frac{y-y_{1}^{\left(s\right)}}{d_{1}^{\prime }}\frac{y-y_{2}^{\left(s\right)}}{d_{2}^{\prime }}\cdots \frac{y-y_{N_{s}}^{\left(s\right)}}{d_{N_{s}}^{\prime }}\right]}^{T} \end{array}$$

and σ*_i_* = σ*_d′_i__*, we obtain

(54) $$\mathrm{Var}(\tilde{x})\geq I_{1,1}^{\prime },\;\mathrm{Var}(\tilde{y})\geq I_{2,2}^{\prime }$$

## 5. Simulation Results

With a number of sensible settings, the feasibility of the proposed schemes is examined via simulation and numerical results. Section 5.1 presents the results of initial position estimation considering the effects of mobility, uncertainty of angle estimation, measurement noise of distance estimation, and number of seeds. Given the initial position estimate and the measurement information, the performance comparisons of three position refinement schemes, the MCMC-based scheme [26], the FLAME method (a fixed fuzzy control algorithm) [35], and the proposed adaptive fuzzy control method, are demonstrated in Sections 5.2 and 5.3.

### 5.1. Initial Position Estimation

**Figure 8.**
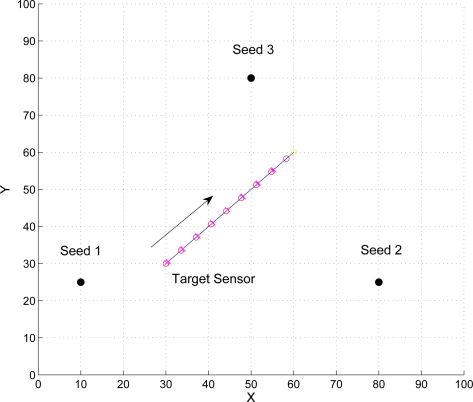
The mobility model of the target sensor.

To evaluate the performance of the proposed approach, we use a custom simulator implemented in Matlab. In the simulation, the TOA and AOA errors are assumed to be Gaussian distributed. Suppose the speed of the signal is 345.6 m/s and the number of samples for particle filtering is 3000. Assume three seeds are with locations ${\text{x}}_{1}^{\left(s\right)}=[10,25]$, ${\text{x}}_{2}^{\left(s\right)}=[80,25]$, and ${\text{x}}_{3}^{\left(s\right)}=[50,80]$, the target mobile sensor is located in a square with side length *l* = 100 m, moving from [30, 30] to [60, 60] (as depicted in Figure 8), and the broadcast interval of the seeds is 1 second. For geometrical positioning with particle filtering, a proper prior density for generating initial samples can be provided by using the idea in [41] and proper convergence can be achieved with five times of iteration.

Four sets of experiments are conducted to evaluate the effects of the variations of critical parameters on position estimation, such as the effect of mobility, the effect of uncertainty of angle estimation, the effect of measurement noise of distance estimation, and the effect of number of seeds. The results consider the difference between the real position and the estimated position of the target mobile sensors. For comparison, the position estimation using the TDOA technique, the position estimation using the conventional TOA technique, and the estimation accuracy using the proposed hybrid TOA/AOA technique are depicted and the CRLBs with perfect AOA information (*i.e.*, $\sigma_{\phi }^{2}=0$) are provided for assessing the performance of the proposed approach.

#### 5.1.1 The Effect of Mobility

**Figure 9.**
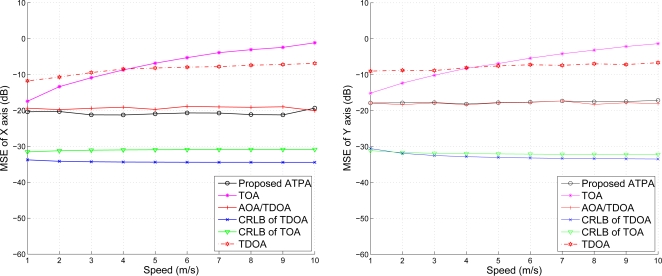
The effect of mobility on position estimation.

For the first set of experiments, we consider the mobility of the target sensor ranging from 1 to 10 m/s. Assuming that the variance of the distance measurement is $\sigma_{d}^{2}=10^{-3}$, Figure 9 shows the position estimation accuracy against the target mobility, which implies that the performance of the TDOA and the proposed TOA/AOA method remain approximately stable regardless of the node moving speed. On the contrary, the estimation error of the conventional TOA method increases with the increasing target speed. Therefore, Figure 9 suggests that ATPA may be suitable for geometrical positioning in situations involving modest mobility.

#### 5.1.2. The Effect of Uncertainty of Angle Estimation

**Figure 10.**
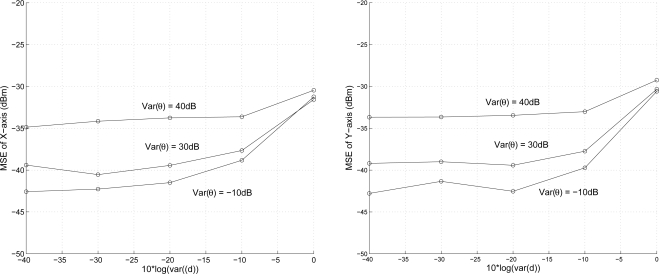
The effect of uncertainty of angle estimation on position estimation accuracy.

Given the target speed 5 m/s and the variance of angle estimation (angle measurement in degree), the second set of experiments investigates the effect of uncertainty of angle estimation on position estimation accuracy with varying the variance of distance measurement ranging from $\sigma_{d}^{2}=10^{-4}∼1$. As expected, Figure 10 illustrates that the proposed scheme achieves better performance with a lower variance of angle estimation. Observe that with a small variance of distance measurement, the angle information dominates the accuracy of position estimation. However, with a larger variance of distance measurement, the localization accuracy is determined by the ranging error.

#### 5.1.3. The Effect of Measurement Noise of Distance Estimation

**Figure 11.**
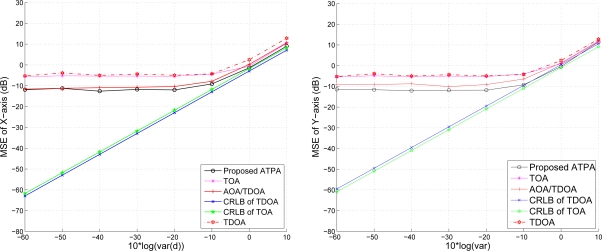
The effect of measurement noise of distance estimation on position estimation.

As shown in Figure 11, given the target speed 5 m/s and the variance of angle estimation $\sigma_{\phi }^{2}=0.1$, the the position estimation error increases with the increasing variance of distance measurement (ranging from $\sigma_{d}^{2}=10^{-4}∼1$). Notice that the CRLB, the performance of the TDOA method, and the performance of the proposed method merge together with a measurement noise σ*_d_* ≥ 1. Therefore, a fundamental problem when locating mobile sensors in a network is to estimate the distance between the seed and the target sensor, since accurate location estimates highly rely on precise distance measurements.

#### 5.1.4. The Effect of Number of Seeds

**Figure 12.**
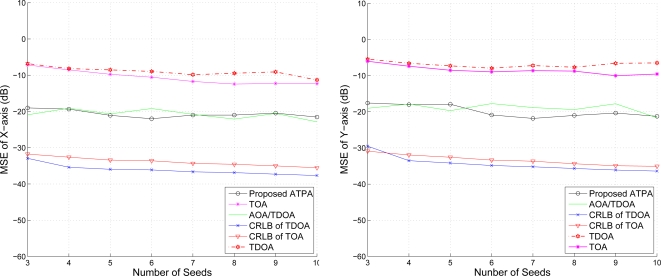
The effect of number of seeds on estimation accuracy.

With the target speed 5 m/s and variance $\sigma_{d}^{2}=10^{-3}$, we vary the number of seeds in the network from 3 to 10. The estimation of position is shown in Figure 12, which shows the accuracy of the position estimate. The performance improves along with the number of seeds. However, like the TDOA approach, the improvement is not significant (especially when number of seeds is greater than 5). This suggests that even a low number of seeds can also achieve good estimation accuracy.

Compared with Figure 9, given 3 seeds with random locations and the speed at 5 m/s, the estimation accuracy of Figure 12 is much lower than the one in Figure 9. This is due to the quality of the TOA measurements. Therefore, the number of TOA measurements for position estimation (*i.e.*, the number of seeds chosen for the measurement) should be dynamically adjusted based on the estimated distance between the target mobile sensor and the seeds in order to reduce location error.

### 5.2. Refinement Schemes: MCMC *vs.* Adaptive Fuzzy Control

Because the particle filter looses diversity in the samples, the Metropolis-Hastings (M-H) algorithm [26] may be used to generate new samples and provide improved estimation accuracy. The basic idea of the M-H algorithm is to simulate an ergodic Markov chain whose samples are asymptotically distributed according to the target probability distribution π(·) and use a candidate proposal distribution ζ(*x_k_*(*i*), ·) to select the candidate of the current state independently with the acceptance probability given by

(55) $$\alpha (x_{k}(i),x_{k}^{\prime }(i))=\text{ min }\left\{1,\frac{\pi (x_{k}^{\prime }(i))\zeta (x_{k}^{\prime }(i),x_{k}(i))}{\pi (x_{k}(i))\zeta (x_{k}(i),x_{k}^{\prime }(i))}\right\}$$

Therefore, instead of using a centralized accumulator host to adjust sensor locations, applying the Markov chain Monte Carlo (MCMC) method on each estimated sensor right after the location estimation allows estimation error to be reduced in a distributed way. Here we summarize the M-H algorithm (a MCMC-based scheme) with the initial value *x*_0_(*i*) in Table 3. In our simulations, the proposal density ζ(*x_k_*(*i*), ·) is composed of the added noise and the current samples generated from particle filtering. The distribution of the noise is 𝒩 (0, σ_ε^2^_) with $\sigma_{ɛ}^{2}=0.1$. From the work in [42], it recommends that if the proposal density is normal, then the acceptance rate should be around 0.45 for the random walk chain. Thus, we adjust the parameters to achieve an acceptance rate of 0.4 to 0.5. The performance of location adjustment by applying a few MCMC steps is reported in the following subsections.

**Figure 13.**
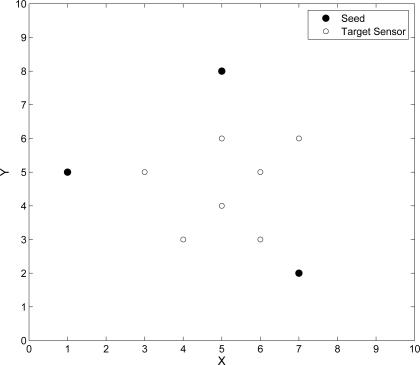
The locations of seeds and unknown target sensors.

Two sets of experiments are applied to evaluate the performance of adaptive fuzzy control and the performance of MCMC technique when using TOA/AOA information, and those only using TOA information to adjust position estimation, respectively. In the simulation, the measurement errors are assumed to be Gaussian random variables. As shown in Figure 13, the reference positions of the three seeds are located with symbols ‘•’ and the true positions of the seven unknown target sensors are located with symbols ‘○’. Incorporating the error analysis in [20,35], the average estimation error *P_err_* can be calculated as follows:

(56) $$P_{\mathrm{err}}=\frac{\sum_{i=1}^{n}\sqrt{{\left({\hat{x}}_{i}-x_{i}\right)}^{2}+{\left({\hat{y}}_{i}-y_{i}\right)}^{2}}}{n}$$

where *n* is the number of unknown target sensors, (*x̂_i_, ŷ_i_*) and (*x_i_, y_i_*) are the estimated position and the actual position of sensor *i*, respectively. Note that in the experimental illustration, the line with the label “Particle Filter” represents the initial position estimate in Phase II. The “N” in the legend denotes the iteration number of fuzzy control. The performance comparisons of these two refinement schemes are depicted in the following subsections.

#### 5.2.1. MCMC *vs.* Adaptive Fuzzy Control (Using TOA Information)

**Figure 14.**
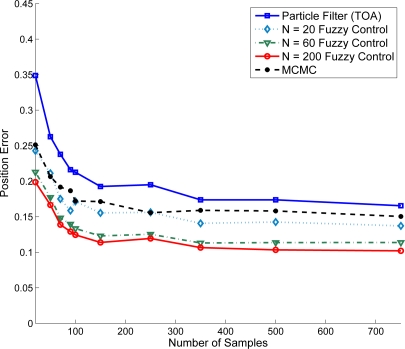
Performances of the MCMC scheme and the adaptive fuzzy control (using TOA information) with varying the number of iterative training.

Given variance of distance estimation $\sigma_{d}^{2}=0.01$ and the number of samples of particle filter (ranging from 25 ∼ 750 samples/area), Figure 14 shows the location adjustment of the target sensor using TOA information. Referring to the line with label - Particle Filter (TOA), it suggests that a better initial position estimate in Phase II may be obtained with a larger sample size. Based on the initial estimation, Figure 14 further shows the improvement of positioning performance when applying the refinement schemes. Observe that the performance of the adaptive fuzzy control with an increase in the number of iterative training may be superior to that of the MCMC method with a smaller sample size. This is attributed to the fact that the number of MCMC samples may have an influence on the particle set’s quality [43]. For the proposed fuzzy control method, the fitting for training data is good.

#### 5.2.2. MCMC *vs.* Adaptive Fuzzy Control (Using AOA/TOA Information)

**Figure 15.**
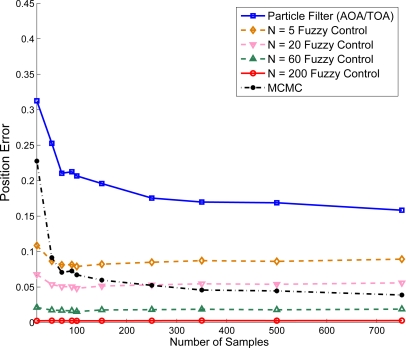
Performance comparison with varying the number of iterative for adaptive fuzzy control (using AOA/TOA information).

Suppose that the target sensor receives AOA and TOA information to estimate its own coordinate. The AOA measurement noise is assumed to be a Gaussian random variable and AOA is measured with degree information. Similar to the results described in Figure 14, Figure 15 show that, given the variance of distance $\sigma_{d}^{2}=0.01$ and variance of angle $\sigma_{\phi }^{2}=0$, the position estimation error is suppressed with increasing the number of samples (ranging from 25 ∼ 750 samples/area). Notice that compared with the MCMC method, the adaptive fuzzy control using AOA and TOA information has less position estimation error. Moreover, compared with Figure 14, Figure 15 shows that incorporating accurate angle information may help tackle the localization problem in addition to distance measurements. Hence, one possible way to approach network localization is to include other measurements such as angle information and heading information [39] in order to suppress the computational complexity.

**Figure 16.**
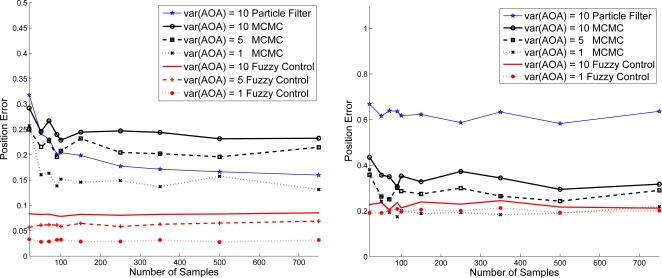
The estimation performance with varying the variance of angle information; $\sigma_{d}^{2}=0.01$ (left) and $\sigma_{d}^{2}=0.5$ (right).

In Figure 16, given the variance of distance $\sigma_{d}^{2}=0.01$ and $\sigma_{d}^{2}=0.5$, we explore the estimation performance with varying the variance of angle information. Notice that as shown in Figure 16 (left), the proposed adaptive fuzzy control with moderate noisy angle information may dominate the localization performance under the circumstances of good distance estimation. However, as shown in Figure 16 (right), even with the moderate noisy angle information, the noisy distance information may have the predominant influence and degrade the estimation performance due to the performance loss caused by measurement uncertainties and propagation environments. Therefore, the MCMC and the proposed adaptive fuzzy control have roughly the same estimation performance in this scenario.

### 5.3. Refinement Schemes: Adaptive Fuzzy Control *vs*. Fixed Fuzzy Control

**Figure 17.**
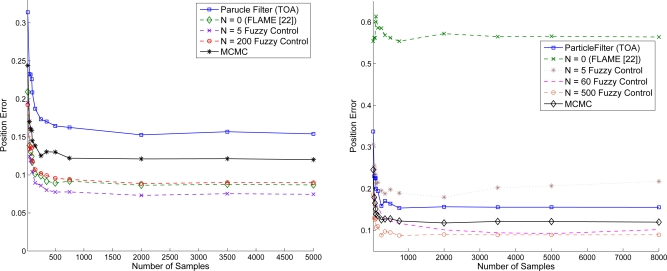
Performance comparison of the ATPA and the FLAME algorithms.

This set of experiment compares the estimation performance of the ATPA with an adaptive fuzzy control scheme and the FLAME with a fixed fuzzy control method [35]. Simulation study is conducted to show that the performance of the FLAME approach is superior to those in [29,44,45]. Thus, the FLAME heuristic may provide a good way to benchmark the performance of ATPA scheme. Here we examine the estimation performance with two sets of fuzzy controller parameters. In Figure 17 (left), appropriate initial settings of controller parameters with fixed fuzzy rules may sensibly improve the estimation accuracy. Observe that the performance gap between these two methods is small. In contrast, as shown in Figure 17 (right), inappropriate initial parameter settings with fixed fuzzy design rules may make the estimation performance even worse since the parameter settings may vary from different scenarios. Thus, because of the lack of learning process, the estimation accuracy with a fixed fuzzy control method may highly depend on the parameter settings and fuzzy logic. On the other hand, even with inappropriate initial settings, the proposed adaptive fuzzy control scheme may still converge the estimation behavior and suppress the estimation error. Therefore, the ATPA approach owns adaptive flexibility when dealing with uncertainty in position estimation.

### 5.4. The Effect of Mobility on the ATPA

**Figure 18.**
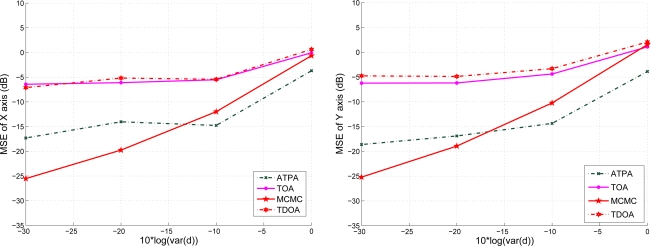
The effect of mobility on position estimation (using TOA information); position error of the x-coordinate (left) and position error of the y-coordinate (right).

**Figure 19.**
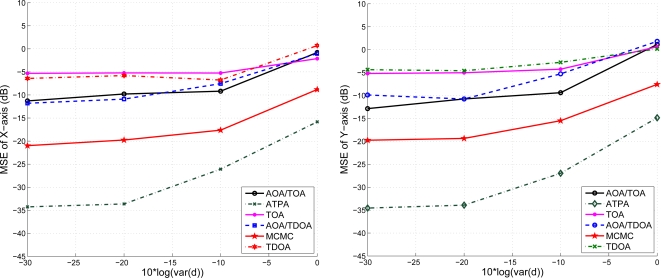
The effect of mobility on position estimation (using AOA/TOA information); position error of x (left) and position error of y-axis (right).

Given the mobility model of the target sensor as described in Figure 8 with speed 5 m/s, Figure 18 shows the position error of x-axis and y-axis with varying the variance of distance when using TOA information. We compare the estimation performances using the proposed positioning method with fuzzy control and that with MCMC. Note that the MCMC position accuracy is better than that of the fuzzy control with low distance variance in this example, but the fuzzy control scheme is computational cheap compared with the MCMC algorithm. With a larger distance variance, the positioning accuracy of the fuzzy control is better than that of the MCMC. In Figure 19, with the AOA and TOA information and the variance of AOA estimation $\sigma_{\phi }^{2}=0.1$, the proposed position system effectively reduces the position error. Compared with the positioning performances with only TOA information (described in Figure 18), the MCMC scheme and the fuzzy control method have superior positioning performances with TOA and AOA information (described in Figure 19), which suggests that the AOA information may help to suppress the estimation error due to the noisy measurements (ranging from −10 ∼ 0 dB).

## 6. Conclusions

This paper describes a distributed AOA-aided TOA positioning algorithm in mobile wireless sensor networks. The algorithm exploits the information flow while coping with distributed signal processing and the requirements of network scalability. Once the estimation procedure and communication protocol are performed, all mobile sensors obtain adequate observations and localize themselves automatically by receiving the periodic broadcasts from the seeds. For the accuracy of initial estimation, the simulations show that the proposed ATPA approach is comparable with the TDOA technique. For the accuracy of refinement, compared with the MCMC scheme and the FLAME algorithm, the proposed ATPA approach provides adaptive flexibility and robust improvement in estimation with moderate noisy measurements. The comparison with the MCMC and the fuzzy control method shows that trade-offs are found between model complexity, estimation accuracy, and sensible model description in real systems. Future plans will involve generalizing the methods to perform actual measurements to evaluate the performance of the proposed positioning system in ubiquitous computing environments.

## Figures and Tables

**Table 1.** The Particle Filtering Methodology.

1.	*Initialization*: Generate a set of random samples *x_k_*(*i*), *i* = 1, 2, . . ., *N_PF_* from the prior density at time *k* = 0. Each sample of the state vector is a ‘particle’.
2.	*Prediction*: Each random sample is passed through the state equation to obtain samples from the prior density at time *k* + 1. Thus ${\hat{x}}_{k+1}(i)=\Phi x_{k}(i)+\Gamma \lambda_{k}(i)$ where *λ_k_*(*i*) is a sample drawn from the probability density function of the system noise, Φ is related to the mobility model, and Γ is an identity matrix.
3.	*Measurement Update*: The weights of the likelihood function *p*(*z_k+_*_1_|*x̂*_*k*+1_(*i*)) are updated for each sample in the random set *i =* 1, 2, *. . ., N_PF_* and the normalized weights are $\xi_{k+1}(i)=\frac{p(z_{k+1}|{\hat{x}}_{k+1}(i))}{\sum_{j=1}^{N_{\mathrm{P F}}}p(z_{k+1}|{\hat{x}}_{k+1}(j))}$ for each sample.
4.	*Re-sampling*: Take *N_PF_* samples with replacement from the random sample set *x̂*_*k*+1_(*i*)), *i =* 1, 2, . . ., *N_PF_*, to generate the new sample set *x_k_*_+1_(*i*).
5.	*Position*: The best single estimate of the position is the mean of *x*_*k*+1_(*i*), $\bar{x_{k}}$.

**Table 2.** Representation of fuzzy rules.

R1 (Rule 1):	**IF**$x_{1}^{\mathrm{diff}}$ is N (negative) **AND**$x_{2}^{\mathrm{diff}}$ is N (negative)
	**THEN** distance adjustment is the value of *w*_1_.
R2 (Rule 2):	**IF**$x_{1}^{\mathrm{diff}}$ is P (positive) **AND**$x_{2}^{\mathrm{diff}}$ is N (negative)
	**THEN** distance adjustment is the value of *w*_2_.
R3 (Rule 3):	**IF**$x_{1}^{\mathrm{diff}}$ is N (negative) **AND**$x_{2}^{\mathrm{diff}}$ is P (positive)
	**THEN** distance adjustment is the value of *w*_3_.
R4 (Rule 4):	**IF**$x_{1}^{\mathrm{diff}}$ is P (positive) **AND**$x_{2}^{\mathrm{diff}}$ is P (positive)
	**THEN** distance adjustment is the value of *w*_4_.

**Table 3.** The Metropolis-Hastings Algorithm.

1.	Set *k* = 0 and repeat for *x_k_*(*i*), *i* = 1, 2, . . ., *N_PF_*. *N_PF_* is the number of samples for particle filtering.
2.	Draw *x′_k_*(*i*) from the proposal density *ζ*(*x_k_*(*i*), ·).
3.	Set *u* to a draw from a *U* (0, 1) distribution.
4.	Acceptance probability: $\alpha (x_{k}(i),x_{k}^{\prime }(i))=\text{ min }\left\{1,\frac{\pi (x_{k}^{\prime }(i))\zeta (x_{k}^{\prime }(i),x_{k}(i))}{\pi (x_{k}(i))\zeta (x_{k}(i),x_{k}^{\prime }(i))}\right\},$ where *π*(·) is the target density from which samples are desired.
5.	**If** (*u* ≤ Acceptance Probability)
	accept proposal and set *x*_*k*+1_(*i*) = *x*′*_k_*(*i*).
	**else**
	reject proposal and set *x*_*k*+1_(*i*) = *x_k_*(*i*).
	**end**
6.	Return the values{*x*_*k*+1_(1), *x*_*k*+1_(2), . . ., *x*_*k*+1_(*N_PF_*)} and set *k* = *k* + 1.
